# Floral Guide

**Published:** 1885-01

**Authors:** 


### Floral Guide.

Vick's Illustrated Monthly Magazine makes its monthly visits ever fully freighted with good things about flowers, plants, and vegetables. Any one at all interested in these things can not afford to be without this most excellent journal. Each number contains matter appropriate to floriculture for the month of its issue. The proper culture of plants and flowers require attention throughout the year, and Vick gives specific directions for every detail.

Vick's Floral Guide is also just at hand, a beautiful specimen of journalistic art. It alone is worth far more than the subscription price of the magazine for the year. It is fully and beautifully illustrated.

